# Doxorubicin-Induced Cardiomyopathy: A Preliminary Study on the Cardioprotective Benefits of 7-Hydroxyflavanone

**DOI:** 10.3390/ijms242015395

**Published:** 2023-10-20

**Authors:** Nonhlakanipho F. Sangweni, Kwazi Gabuza, Ruzayda van Aarde, Lawrence Mabasa, Derick van Vuuren, Barbara Huisamen, Reenen Barry, Rabia Johnson

**Affiliations:** 1 Biomedical Research and Innovation Platform (BRIP), South African Medical Research Council, Tygerberg, Cape Town 7505, South Africa; kwazi.gabuza@mrc.ac.za (K.G.); ruzayda.aarde@mrc.ac.za (R.v.A.); lawrence.mabasa@mrc.ac.za (L.M.); rabia.johnson@mrc.ac.za (R.J.); 2 Centre for Cardio-Metabolic Research in Africa, Division of Medical Physiology, Faculty of Medicine and Health Sciences, Stellenbosch University, Tygerberg, Cape Town 7505, South Africa; dvvurren@sun.ac.za (D.v.V.); bh3@sun.ac.za (B.H.); 3 Biopharm, Hamilton 3200, New Zealand; reenen@biopharmnz.com

**Keywords:** flavonoids, cardiotoxicity, doxorubicin, cardioprotection

## Abstract

The therapeutic properties of flavonoids are reported to offer cardioprotective benefits against doxorubicin (Dox)-induced cardiotoxicity (DIC). In the current study, we aimed to investigate the prophylactic properties of 7-hydroxyflavanone (7H), a flavonoid with antioxidative properties, against DIC. An in vitro model of DIC was established by exposing H9c2 cardiomyoblasts to Dox for 6 days. Similarly, cells were also co-treated with 7H to assess its ability to mitigate DIC. The data obtained indicate that 7H, as a co-treatment, alleviates Dox-induced oxidative stress by enhancing total glutathione content (*p* ≤ 0.001) and superoxide dismutase activity (*p* ≤ 0.001) whilst decreasing ROS (*p* ≤ 0.001), malondialdehyde production (*p* ≤ 0.001) and the secretion of interleukin-6 (*p* ≤ 0.001). The data also showed an improvement in mitochondrial function as shown via enhanced bioenergetics, mitochondrial membrane potential, and PGC1-alpha (*p* ≤ 0.05) and pAMPK (*p* ≤ 0.001) expression. The cardioprotective potential of 7H was further highlighted by its ability attenuate Dox-induced caspase 3/7 activity (*p* ≤ 0.001), apoptosis (*p* ≤ 0.001) and necrosis (*p* ≤ 0.05). In conclusion, our findings demonstrated the cardioprotective benefits of 7H and thus suggests that it could be a suitable candidate cardioprotective agent against DIC.

## 1. Introduction

The clinical limitations of the anthracycline (ATC) and doxorubicin (Dox), were first described in a cancer clinical trial in 1970 [1]. In this study, the cytotoxic effect of Dox not only affected the malignant tumors but also induced adverse reactions in neighboring tissues, such as the myocardium [1]. Briefly, 23 out of 47 patients who had originally presented with normal cardiac function were found to have developed transient electrocardiographic abnormalities a week after treatment cessation [1]. However, the authors argued that the chemotherapeutic effects of Dox were too potent to ignore despite the observed cardiotoxic side effects. To date, several clinical and preclinical studies have consistently demonstrated the harsh effects of Dox administration on the heart, either during chemotherapy or once treatment has concluded [2,3,4,5,6]. Nonetheless, Dox remains a first-line chemotherapeutic drug that is routinely scheduled for and administered to cancer patients. Perhaps the argument for its continued use is that we simply cannot ignore its potent anti-cancer properties, but the conundrum is that today’s cancer survivors may very well be tomorrow’s cardiovascular disease (CVD) patients.

Indeed, the burden of DIC and its unclear etiology has led to a plethora of research which aims to identify novel therapeutic options and current therapies that can be repurposed as prophylactics for DIC. The primary focus in these studies entails understanding how alternative therapies can mitigate DIC via the targeting of any of the multiple mechanisms that are associated with the disease pathophysiology [7,8,9]. For instance, Dox’s ability to inhibit ferritin whilst increasing the expression of transferrin triggers cardiac iron overload and facilitates the production of reactive oxygen species (ROS) and the destruction of cellular membranes via lipid peroxidation and impaired antioxidant signaling. The imbalance in redox substances is further driven via Dox’s high affinity to cardiolipin and the displacement of coenzyme Q10, which allows Dox to redirect electrons from the electron transport system (ETS) to generate more ROS and depolarize the mitochondria [10,11]. These reactions impair inflammatory and autophagic response, whilst aggravating cardiomyocyte death, which is a necessary step in the development of cardiomyopathy [12,13].

Evidence suggests that the cardioprotective benefits of flavonoids might be useful in reducing the burden of DIC [14]. Indeed, the flavonoids quercetin and luteolin are reported to attenuate DIC by preventing oxidative stress, mitochondrial dysfunction, impaired autophagy and apoptosis in in vitro models of cardiotoxicity [15,16]. Moreover, quercetin, as an adjunct to Dox, increases the antitumor effect of Dox via enhanced p53 and cleaved caspase 3 expression, and decreased cancer cell migration [16,17]. Of interest to this study is the flavonoid 7-Hydroxyflavanone (7H), which exhibits 20S proteasome and aromatase inhibitory properties [18]. The literature shows that proteasome inhibition alleviates cardiomyopathy by improving left ventricular function via the upregulation of atrial natriuretic peptide (ANP), β myosin heavy chain and NRF-2 expression [19] and reduces myocardial infarct size by regulating inflammation, autophagy and antioxidant activity [20]. Although no association has been made with the relevance of 7H and CVDs, the flavanone’s proteasome inhibitory properties and anti-cancer activity warrant a need to explore its usefulness as a cardioprotective agent. Therefore, the current study aimed to assess the prophylactic benefits of 7H against DIC using an H9c2 in vitro model.

## 2. Results

### 2.1. Dose Response of Flavonoids

**Figure 1:**
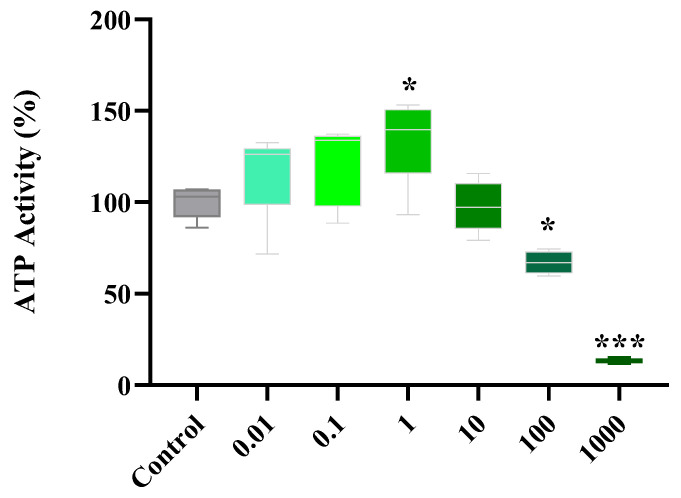
Cytotoxicity screening of 7-Hydroxyflavanone (7H) on the H9c2 cardiomyoblasts. Concisely, cells were treated with various concentrations of 7H for 6 days. A one-way analysis of variance (ANOVA) and a Bonferroni post hoc test were used to analyze the data, which are presented as the mean ± SEM of 4 biological experiments with 6 technical repeats (*n* = 4). Statistical significance is depicted as * *p* ≤ 0.05, *** *p* ≤ 0.001 versus the control.

The ATP assay revealed that 7H, at 0.01–10 µM, had no cytotoxic effects on cardiac cells but, significantly decreased (*p* ≤ 0.001) cardiomyoblast metabolic activity at higher concentrations (100–1000 µM) when compared to that of the control (Figure 1). A comparative analysis across the different concentrations showed that 1 µM had a more potent effect on cardiomyoblast metabolic activity as shown via enhanced ATP levels (*p* ≤ 0.05). As such, all subsequent experiments were performed using a 1 µM 7H concentration.

### 2.2. Doxorubicin Dose Response

**Figure 2:**
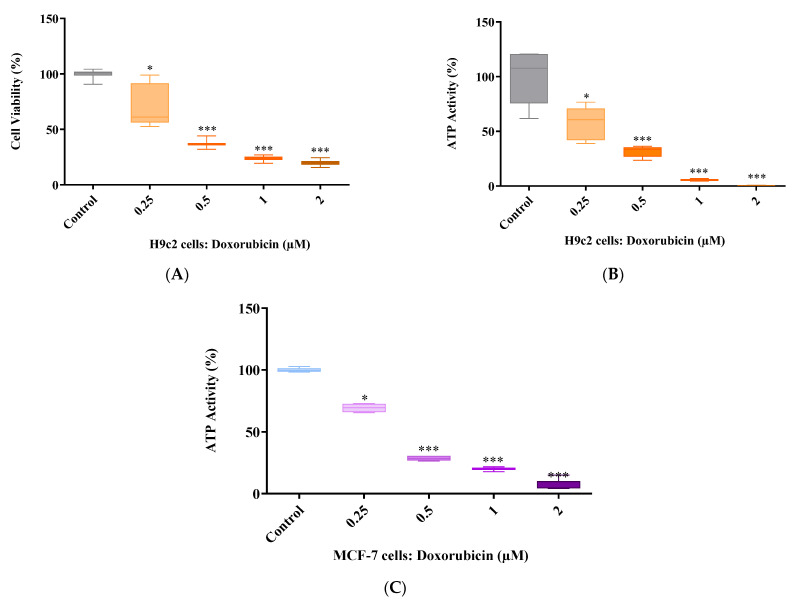
Cytotoxicity screening of doxorubicin (Dox). (**A**) Cell viability in H9c2 cells, (**B**) ATP activity in H9c2 cells and (**C**) ATP activity in MCF-7 cells. H9c2 cardiomyoblasts and MCF-7 breast cancer cells were treated with varying Dox concentrations (0.25, 0.5, 1 and 2 µM) for 6 days. A one-way analysis of variance (ANOVA) and a Bonferroni post hoc test were used to analyze the data, which are presented as the mean ± SEM of 4 biological experiments with 6 technical repeats (*n* = 4). Statistical significance is depicted as * *p* ≤ 0.05 and *** *p* ≤ 0.001 versus the control.

The severity of cardiotoxicity, caused by Dox, relies solely on the concentration being used and the duration of treatment exposure. In a previous study, we reported that a 6-day treatment exposure of 2 µM Dox was sufficient to induce cardiotoxicity without killing 80% of cardiac cells [21]. However, due to batch variation, which resulted in 2 µM Dox killing over 80% of the H9c2 cells, we performed a dose response study to attain the most suitable concentration to induce DIC for the current in vitro study. The results show that 2 µM Dox reduces cardiomyocyte viability to 19% whilst inhibiting ATP activity after a 6-day treatment exposure (*p* ≤ 0.001 versus the control) (Figure 2A,B). Similar findings were also observed for cells treated with 1 µM Dox (*p* ≤ 0.001 versus the control). However, at a dose of 0.5 µM, Dox led to a 63% and 69% reduction in cardiomyocyte viability and ATP activity, respectively (*p* ≤ 0.001 vs. the control) (Figure 2A,B). Although influencing the H9c2 cells’ ATP levels (57%), 0.25 µM demonstrated insufficient cytotoxic effects as indicated by the cardiomyocyte viability of 80% when compared to that of the control (Figure 2A,B). More importantly, cancer cells treated with 0.25 µM Dox presented an ATP activity of 70% after 6 days, which suggests Dox’ lack of potency at this concentration (Figure 2C). However, much like the H9c2 cells, MCF-7 cells treated with 0.5 µM Dox presented an ATP activity of 28%, which was significantly lower than that of the control (*p* ≤ 0.001) (Figure 2C). Moreover, the ATP activity of MCF-7 cells treated with 1 µM and 2 µM Dox was 20% and 6.7%, respectively (Figure 2C). Based on these results, 0.5 µM Dox was selected to be used for all subsequent experiments (Figure 2A–C).

### 2.3. Doxorubicin-Induced Oxidative Stress in Cardiac Cells

**Figure 3:**
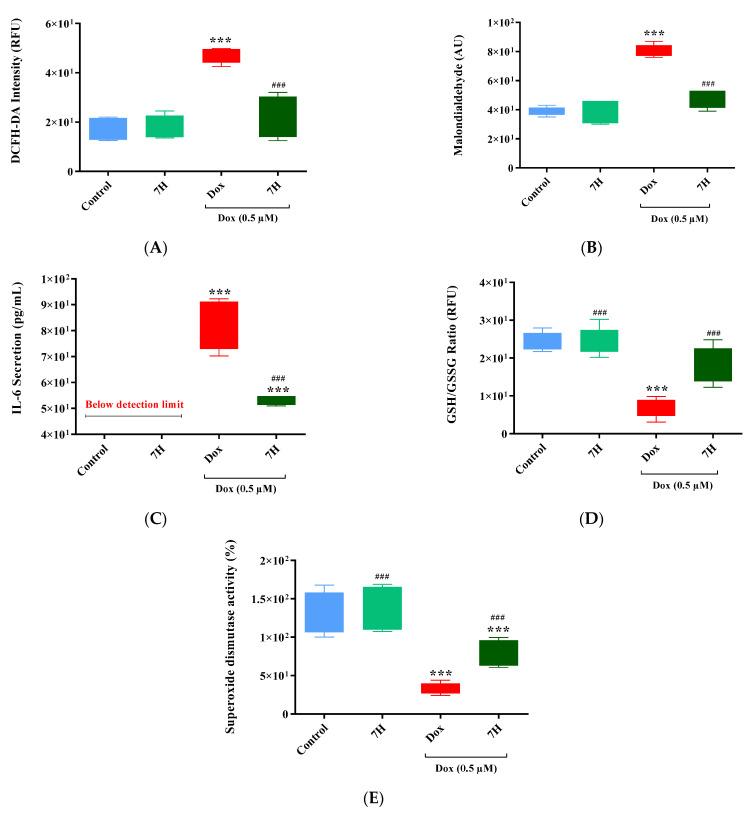
7-Hydroxyflavanone (7H) mitigates doxorubicin (Dox)-induced oxidative stress in H9c2 cells. (**A**) 2,7-dichlorofluorescin diacetate (DCFH-DA, (**B**) lipid peroxidation, (**C**) interleukin-6 (IL-6), (**D**) ratio of reduced glutathione (GSH) to oxidized glutathione (GSSG), and (**E**) superoxide dismutase (SOD). The cells were treated with 7H in the absence or presence of Dox, for 6 days. A one-way analysis of variance (ANOVA) and a Bonferroni post hoc test were used to analyze the data, which are presented as the mean ± SEM of 3 biological experiments with 6 technical repeats (*n* = 3). Statistical significance is depicted as *** *p* ≤ 0.001 versus the control, and ^###^ *p* ≤ 0.001 versus Dox.

In line with previous studies, Dox exposure induced oxidative stress by increasing ROS and lipid peroxide production (*p* ≤ 0.001), and IL-6 secretion in the cardiomyocytes (*p* ≤ 0.001 versus the control) (Figure 3A–C). These results were supported via the reduced endogenous antioxidants, total glutathione (GSH, *p* ≤ 0.001) content and superoxide dismutase activity (SOD, *p* ≤ 0.001) (Figure 3D,E). However, when cardiomyocytes were co-treated with 7H, we observed a significant decrease in prooxidants (ROS and lipid peroxide; *p* ≤ 0.001) and pro-inflammatory marker IL-6 (*p* ≤ 0.001), with a concomitant improvement in SOD and GSH (*p* ≤ 0.001, versus Dox) (Figure 3A–E). These findings are indicative of 7H’s ability to mitigate Dox-induced oxidative damage. 

### 2.4. Mitochondrial Bioenergetics of Cardiac Cells

**Figure 4:**
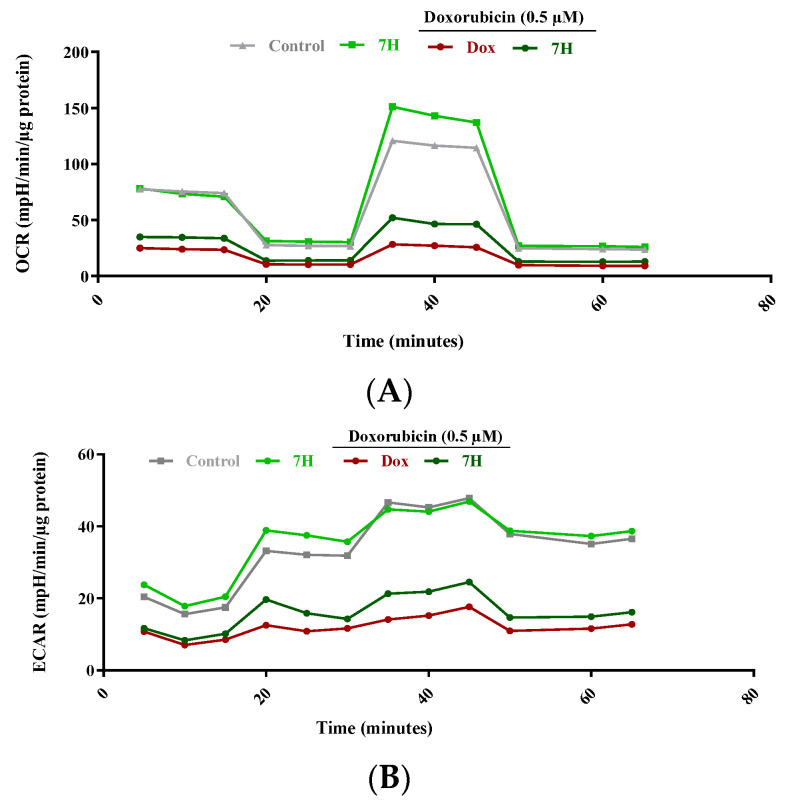
The effect of 7-Hydroxyflavanone (7H) on impaired mitochondrial bioenergetics. (**A**) Oxygen consumption rate (OCR) and (**B**) extracellular acidification rate (ECAR). H9c2 cells were treated with 7H in the absence or presence of Dox, for 6 days. A one-way analysis of variance (ANOVA) and a Bonferroni post hoc test were used to analyze the data, which are presented as the mean ± SEM of 3 biological experiments with 8 technical repeats (*n* = 3).

Several experimental models demonstrate that Dox disrupts the electron transport system to impair mitochondrial function. Much like what is reported in the literature, this study demonstrated a shift in the cardiomyocytes’ real-time oxygen consumption rate (OCR) as could be seen via the significant reduction in basal (*p* ≤ 0.01), ATP-linked (*p* ≤ 0.001) and maximal respiration (*p* ≤ 0.001) in cells treated with Dox when compared to those in the control group (Figure 4A and Table 1). This deterioration in mitochondrial bioenergetics was further highlighted by the drastic reduction in the cells’ extracellular acidification rate (ECAR) and spare respiratory capacity (Figure 4B and Table 1) (*p* ≤ 0.001). The depletion of the cells’ spare respiration may drive affected cells into early senescence and resultant apoptosis. As an estimation of mitochondrial function, the respiratory flux ratios of the cardiomyocytes were significantly decreased by Dox (*p* ≤ 0.001), further reiterating its involvement in impaired bioenergetics (Table 1). However, as an adjunct to Dox, 7H was able to ameliorate mitochondrial bioenergetics by enhancing the cardiomyocytes’ maximal (*p* ≤ 0.01) and ATP-linked respiration, as well as the cells’ spare respiratory capacity (*p* ≤ 0.05). Consequently, mitochondrial function, as demonstrated via enhanced coupling efficiency (*p* ≤ 0.001) and respiratory control (*p* ≤ 0.01), was alleviated via co-treatment with 7H (Table 1).

### 2.5. Cardiac ATP Activity and Mitochondrial Membrane Potential

**Figure 5:**
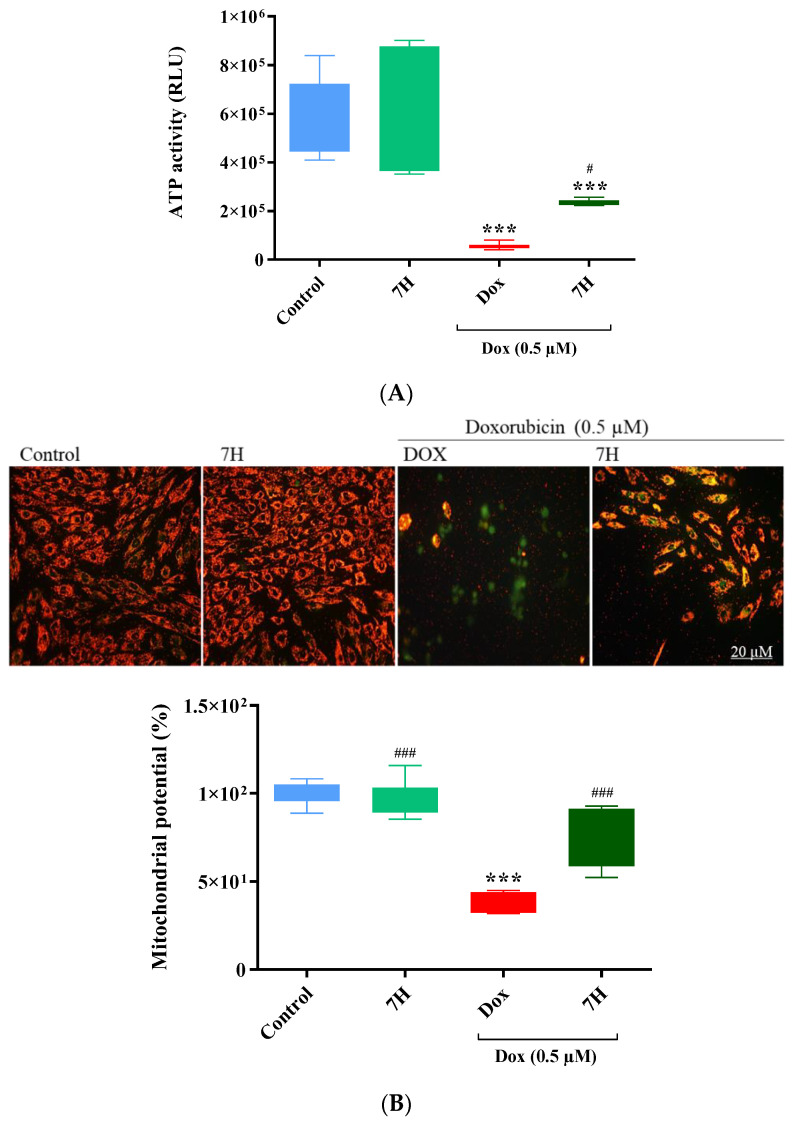
7-Hydroxyflavanone (7H) ameliorates Doxorubicin-induced losses in (**A**) ATP activity and (**B**) mitochondrial membrane potential in H9c2 cells, after 6 days of treatment exposure. H9c2 cells were stained with the JC-1, cationic carbocyanine dye and imaging was performed on a Nikon microscope at a 20× magnification. A one-way analysis of variance (ANOVA) and a Bonferroni post hoc test were used to analyze the data, which are presented as the mean ± SEM of 3 biological experiments with 6 technical repeats (*n* = 3). Statistical significance is depicted as *** *p* ≤ 0.001 versus the control, and ^#^ *p* ≤ 0.05 and ^###^ *p* ≤ 0.001 versus Dox.

The acute inhibition of mitochondrial bioenergetics, due to Dox exposure, stimulates mitochondrial ROS production and leads to insufficient ATP activity, thus depolarizing the mitochondria. Similarly, in this study, impaired oxidative phosphorylation was demonstrated via reduced ATP activity in cardiac cells subjected to Dox exposure (*p* ≤ 0.001, versus the control) (Figure 5A). This was accompanied with increased ROS levels, triggering the permeabilization of the mitochondria, which was represented by a loss in mitochondrial membrane potential (MMP) (*p* ≤ 0.001, versus the control). In contrast, co-treatment with 7H improved the cardiomyocytes’ metabolic activity (*p* ≤ 0.05, versus Dox) and MMP (*p* ≤ 0.001) (Figure 5A,B). These results indicate 7H’s ability to alleviate the mitochondrial damage induced via Dox treatment.

### 2.6. Autophagy and Mitochondrial Proteins

**Figure 6:**
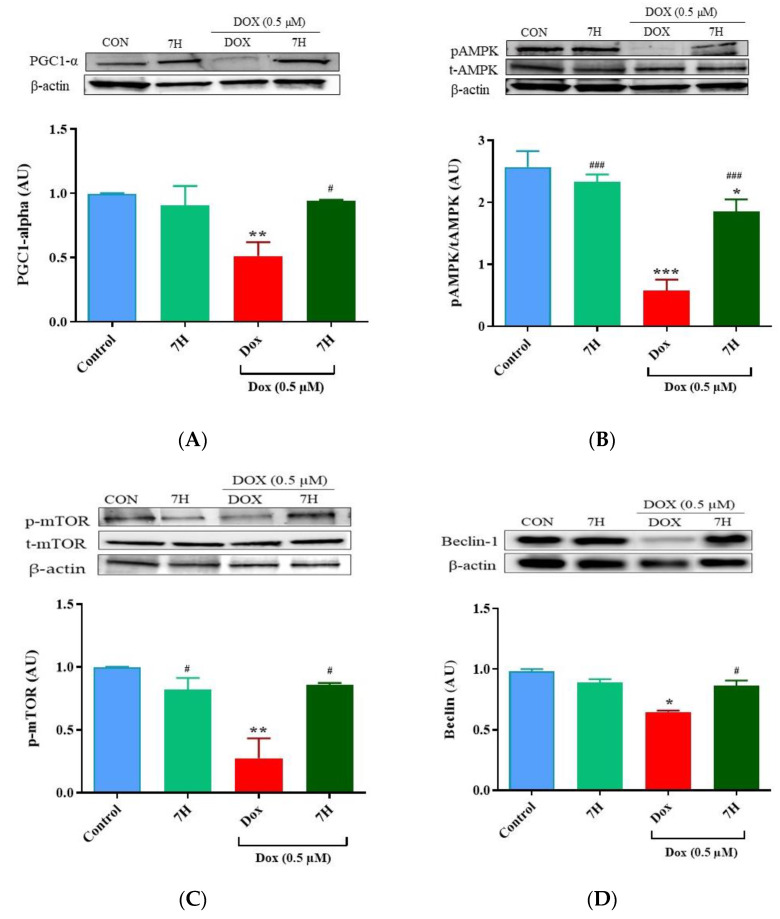

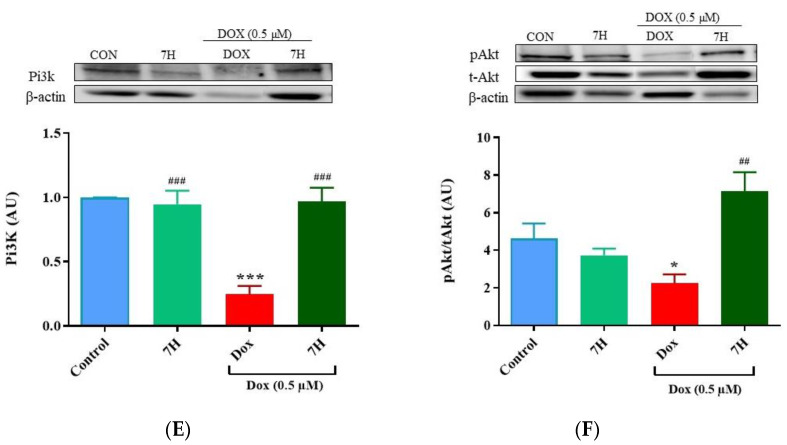
7-Hydroxyflavanone (7H) regulates autophagy. (**A**) Pparg coactivator 1 alpha (PGC-1 α), (**B**) phosphorylated 5′ adenosine monophosphate-activated protein kinase (pAMPK), (**C**) phosphorylated mammalian target of rapamycin (pMTOR), (**D**) Beclin-1, (**E**) Phosphoinositide 3-kinases (PI3K) and (**F**) phosphorylated protein kinase B (pAkt). A one-way analysis of variance (ANOVA) and a Bonferroni post hoc test were used to analyze the data, which are presented as the mean ± SEM of 3 biological experiments with 6 technical repeats (*n* = 3). Statistical significance is depicted as * *p* ≤ 0.05, ** *p* ≤ 0.01 and *** *p* ≤ 0.001 versus the control, and ^#^ *p* ≤ 0.05, ^##^ *p* ≤ 0.01 and ^###^ *p* ≤ 0.001 versus Dox.

Given that impaired mitochondrial function and autophagy are implicated in the development of DIC to drive cardiomyocyte injury by accelerating apoptosis, we quantified the expression of proteins involved in these biochemical processes. Our findings showed a significant reduction in the expression of PGC1-α (*p* ≤ 0.05), pAMPK (*p* ≤ 0.001), pMTOR (*p* ≤ 0.05) and Beclin-1 (*p* ≤ 0.01) in the cardiomyocytes treated with Dox relative to that in the control group (Figure 6A–E). Similarly, a reduction in the expression of PI3K (*p* ≤ 0.001) and pAkt (*p* ≤ 0.05) was also observed in these cells (Figure 6E,F). However, when co-treated with 7H, an increase in the expression of autophagy-related proteins (pMTOR (*p* ≤ 0.05) and Beclin-1 (*p* ≤ 0.05)) was observed, indicating the flavonoids’ ability to mitigate the impaired autophagic response following Dox exposure. This improvement was probably driven via the enhanced expression of proteins involved in mitochondrial bioenergetics (PGC1-α (*p* ≤ 0.05) and pAMPK (*p* ≤ 0.001)) after being co-treated with 7H (Figure 6A,B). Owing to alleviated autophagy and mitochondrial function, an increase in cardiomyocyte viability, as demonstrated via the enhanced expression of PI3K (*p* ≤ 0.001) and pAkt (*p* ≤ 0.001), was observed following co-treatment with 7H (Figure 6E,F).

### 2.7. Doxorubicin-Induced Apoptosis in Cardiac Cells

**Figure 7:**
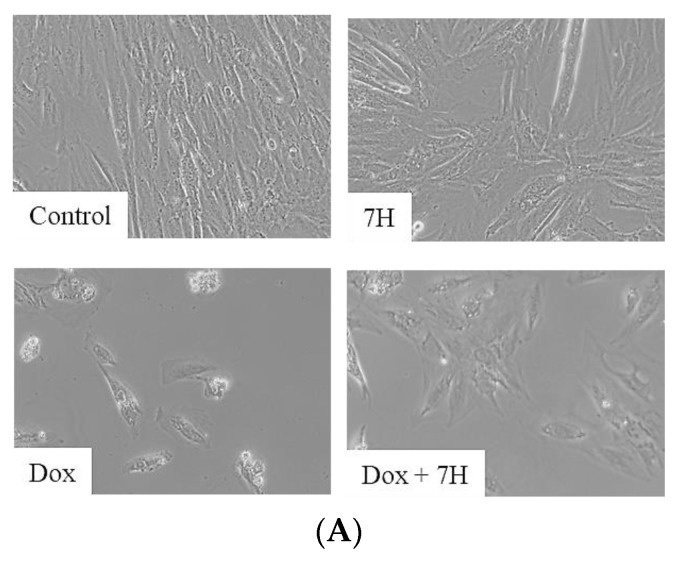

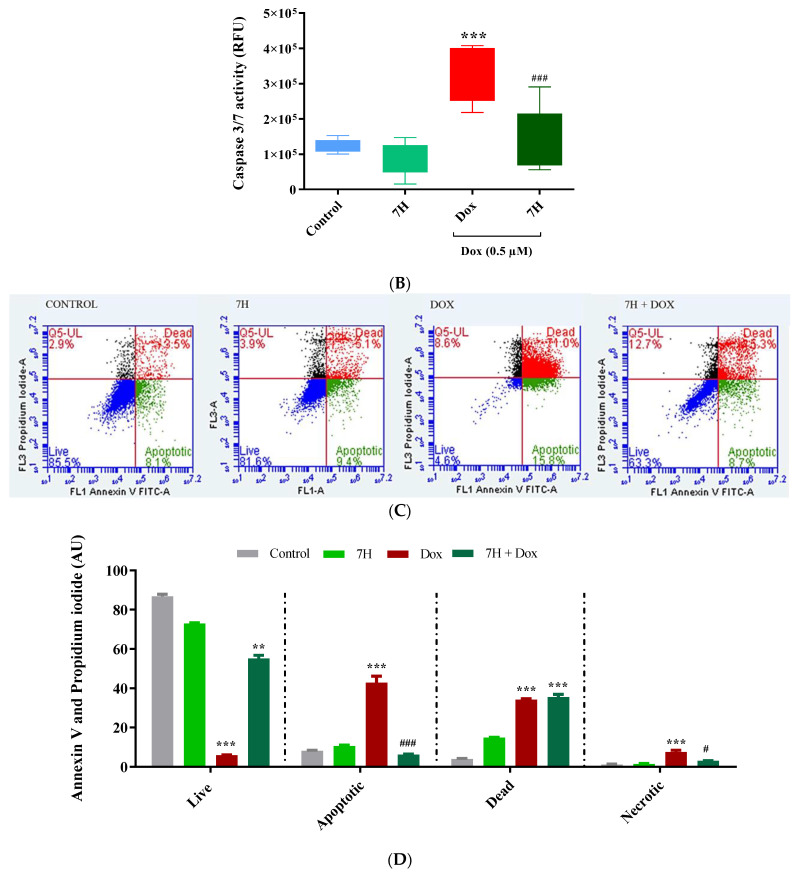
7-Hydroxyflavanone (7H) attenuating doxorubicin (Dox)-induced cell death. (**A**) Bright-field microscopy at 20× magnification, scale bar: 20 μm (**B**) caspase 3/7 activity, (**C**) flow cytometry, and Annexin V and propidium iodide staining, and (**D**) quantification of flow cytometry data. The H9c2 cells were treated with 7H, in the absence or presence of Dox, for 6 days. A one-way analysis of variance (ANOVA) and a Bonferroni post hoc test were used to analyze the data, which are presented as the mean ± SEM of 3 biological experiments with 8 technical repeats (*n* = 3). Statistical significance is depicted as ** *p* ≤ 0.01 and *** *p* ≤ 0.001 versus the control, and ^#^ *p* ≤ 0.05 and ^###^ *p* ≤ 0.001 versus Dox.

A noticeable reduction in the quantity of cardiomyocytes treated with Dox, relative to that in the control, was observed as demonstrated via bright-field microscopy (Figure 7A). This reduction was most likely triggered by damaged mitochondria and the resultant increase in caspase 3/7 activity (*p* ≤ 0.001) (Figure 7A). The reduction in viable cells after Dox exposure was further demonstrated via an increase in early (*p* ≤ 0.001) and late apoptosis (*p* ≤ 0.001), and necrosis (*p* ≤ 0.001) (Figure 7B,C). In contrast, 7H, as an adjunct to Dox, mitigated cardiomyocyte cell death by reducing the activity of caspase 3/7 (*p* ≤ 0.001, versus Dox), which led to an overall reduction in apoptotic (*p* ≤ 0.001, versus Dox), dead (*p* ≤ 0.001, versus Dox) and necrotic cells. These results are indicative of 7H’s cardioprotective potential and were further confirmed via the significant increase in cardiomyocyte viability (*p* ≤ 0.001, versus Dox) (Figure 7A–D).

### 2.8. Breast Cancer Cells: Antioxidant Activity and Caspase 3/7

**Figure 8:**
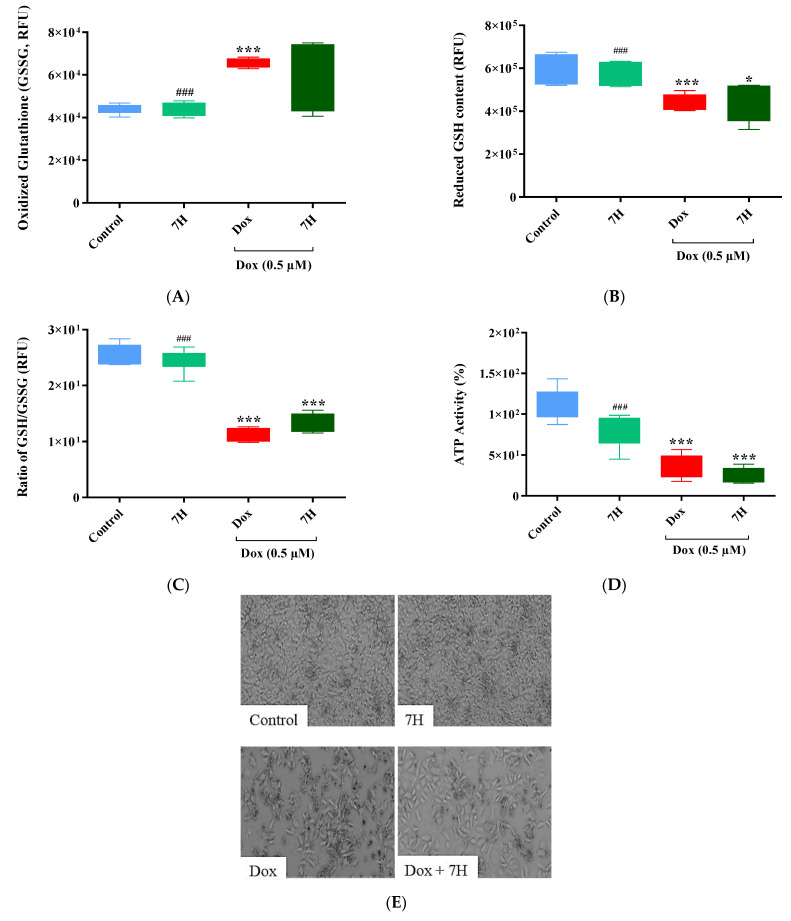

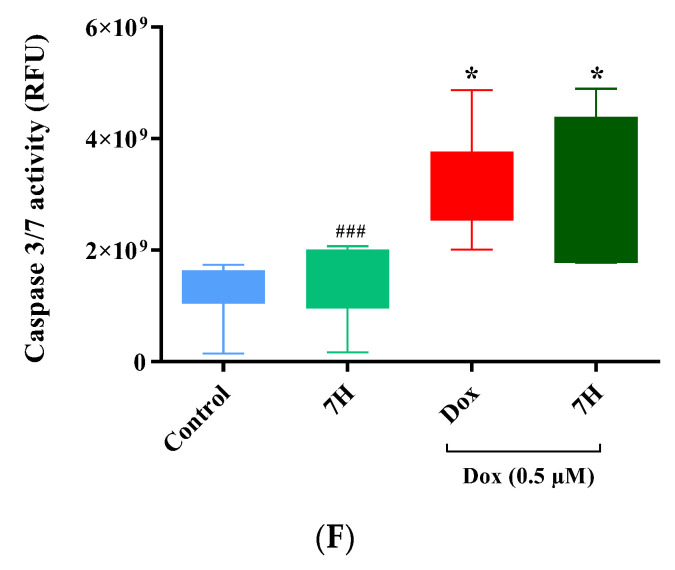
7-Hydroxyflavanone (7H) attenuating doxorubicin (Dox)-induced mitochondrial damage. (**A**) Oxidized glutathione (GSSG), (**B**) reduced glutathione (GSH), (**C**) total glutathione (ratio of GSH/GSSG), (**D**) ATP activity, (**E**) bright-field microscopy at 20× magnification, scale bar: 20 μm and (**F**) caspase 3/7 activity. The MCF-7 cells were treated with 7H in the absence or presence of Dox, for 6 days. A one-way analysis of variance (ANOVA) and a Bonferroni post hoc test were used to analyze the data, which are presented as the mean ± SEM of 3 biological experiments with 6 technical repeats (*n* = 3). Statistical significance is depicted as * *p* ≤ 0.05 and *** *p* ≤ 0.001 versus the control, ^###^ *p* ≤ 0.001 versus Dox.

Numerous therapeutic compounds have been screened for their cardioprotective benefits against Dox-induced cardiotoxicity. At first, these compounds demonstrate potent efficacy in offering cardioprotection; however, these benefits are often short-lived as these compounds may potentially limit the efficiency of chemotherapeutic drugs when co-administered. Hence, the efficacy of Dox, when used in combination with 7H, was investigated on the MCF-7 breast cancer cells. A depletion in the antioxidant defenses of cancer cells was observed following 6-day treatment exposure with Dox. This was represented by a reduction in GSH content (*p* ≤ 0.001 vs. control) with a concomitant increase in GSSG levels (*p* ≤ 0.001 vs. control) (Figure 8A–C). This consequently led to a significant reduction (*p* ≤ 0.001) in the metabolic activity of the cancer cells when compared to that of the control (Figure 8D). Interestingly, co-treatment with 7H had no significant effect on Dox-induced caspase 3/7 activity and reduced total GSH and ATP levels relative to those of cells treated with Dox alone (Figure 8C,E,F).

## 3. Discussion

The burden of DIC has led to an increasing amount of research focusing on its prevention via targeting the mechanisms that are frequently implicated in its development [15,22,23,24]. Amongst these mechanisms are oxidative stress, mitochondrial dysfunction, autophagy and apoptosis, all of which have been shown to facilitate the onset and progression of DIC [25,26]. Evidently, in the current study, Dox exposure stimulated oxidative damage by overproducing ROS and MDA in the cardiomyoblasts. As a result, Dox exhausted the cells’ endogenous antioxidant levels by decreasing the ratio of GSH/GSSG and SOD activity. However, as an adjunct to Dox, the flavonoid 7H was able to mitigate Dox-induced oxidative damage by ameliorating the cardiomyocytes’ antioxidant capacity (GSH/GSSG and SOD), which led to an even further reduction in hydroxyl radicals, lipid peroxides and IL-6 secretion. These findings are in line with previous observations which highlight the importance of regulating redox-mediated mechanisms in the management of DIC [5,27]. Indeed, in a previous study, vanillin, which is a bioactive polyphenolic agent found in curcumin with anti-inflammatory and antioxidative properties, was shown to mitigate DIC by preventing oxidative stress in H9c2 cells, which led to a decrease in DNA damage and apoptosis. The authors further showed that this polyphenolic compound does not impede the antineoplastic properties of Dox when tested on U20S osteosarcoma cells [5]. Similarly, when we investigated the effect of 7H on the efficacy of Dox to disrupt the antioxidant defense system of the MCF-7 breast cancer cells, we observed no significant changes in the ratio of GSH/GSSG, which was largely driven by the reduction in GSH content and increased GSSG levels in the cancer cells that were either co-treated with Dox plus 7H or treated with Dox alone. The relevance of GSSG in oxidative stress has been previously described by Nur et al., where the increased efflux of GSSG depletes intracellular antioxidant defenses [28]. This, therefore, suggests that the increased GSSG levels observed in this study may aid in stimulating cancer cell oxidative damage and, by doing so, promote tumor regression.

The heart’s innately lower antioxidant status compared to that of other organs makes it readily susceptible to Dox-induced intracellular toxicity, which results in the damage of various organelles such as the mitochondria [12]. Doxorubicin’s accumulation in the mitochondria is enabled via its high affinity to cardiac mitochondria, which are easily targeted by Dox due to Dox’s high affinity to cardiolipin, which is a mitochondrial membrane-bound phospholipid that facilitates its accumulation within the mitochondria [13]. In the current study, Dox weakened cardiac mitochondrial bioenergetics by impeding the cells’ basal and maximal respiratory capacity, which led to a reduction in the respiratory flux ratios, thereby compromising mitochondrial function. These findings were confirmed via the reduced protein expression of PGC-1α and pAMPK, which consequently impaired cardiac energy homeostasis. Indeed, intact perfused hearts exposed to 2 µM Dox were previously shown to have decreased pAMPK activity, and thus supported the involvement of Dox in impaired myocardial bioenergetics [12]. In contrast, cardiomyocytes co-treated with 7H presented a significant improvement in mitochondrial bioenergetics and function, which was supported via the enhanced expression of pAMPK and PGC-1α, and increased MMP. These findings are especially important as the increased expression of PGC-1α in cardiomyocytes is likely to stimulate a compensatory response to preserve mitochondrial integrity, by mitigating the loss in MMP. While the efficacy of 7H as a potential cardioprotective agent against DIC is appealing, equally important is ensuring that the efficacy of Dox as a chemotherapeutic drug is sustained. Hence, we assessed the degree of cancer cell damage following co-treatment with Dox plus 7H. The results revealed no significant changes in the metabolic status of the MCF-7 cells co-treated with 7H and Dox. Similarly, while 7H had a noticeable effect on the activity of caspase 3/7 in the MCF-7 cells, these changes were statistically insignificant when compared to the modifications induced by Dox in these cells. These results thus suggest that while 7H exhibits promising cardioprotective benefits when used as an adjunct to Dox, it is likely to influence the chemotherapeutic potential of Dox, albeit insignificantly.

In the context of mitochondrial toxicity, autophagic responses are said to be stimulated to assist with the removal of damaged mitochondrial structures to preserve cellular homeostasis. However, during Dox treatment, autophagy is dysregulated, where Dox either accelerates this process, to reduce myocardial mass [29], or shuts it down completely to trigger cardiac oxidative damage [30]. Similarly, in this study, Dox dysregulated autophagy by reducing the expression of autophagy-related proteins in the cardiomyocytes. However, this process was mitigated in cardiomyocytes that were co-treated with 7H. Evidently, 7H enhanced the phosphorylation of MTOR and increased the expression of Beclin-1-1 in these cells. Since pMTOR partially inhibits the downstream signaling of Beclin-1-1, we speculate that the observed increased expression of these two proteins, to drive autophagy, was attributed to the enhanced phosphorylation of AMPK. Indeed, the literature indicates that AMPK-mediated autophagy is initiated under pathophysiological conditions to protect the heart from a pressure overload [31]. Furthermore, Xu et al. showed that initiating autophagy in a controlled manner during periods of Dox exposure could alleviate the burden of DIC by protecting the heart from Dox-mediated cell death and resultant cardiac damage [15].

Considering the mechanistic processes mentioned above, it is quite evident that they collectively contribute to Dox-induced cell death, which is recognized as a necessary process for the onset of left ventricular remodeling and dysfunction [12,32]. Indeed, the observed loss in MMP releases mitochondrial-bound pro-apoptotic proteins which facilitate the activation of caspase 3/7 activity to induce cell death. These findings were confirmed via bright field microscopy, which revealed a 90% loss in cellular viability. To further confirm the flavonoids’ cardioprotective benefits, our results showed, that as an adjunct to Dox, 7H was able to mitigate Dox-induced apoptosis by decreasing caspase 3/7 activity, which vastly alleviated the degree of apoptosis. Additionally, the protein expression of pro-survival proteins PI3K and pAkt were also increased via the co-treatment.

## 4. Methods

### 4.1. Materials and Reagents

The Annexin V and fluorescein conjugate (FITC annexin V) was purchased from Invitrogen (Carlsbad, CA, USA). Propidium iodide (PI), 3-(4,5-dimethylthiazol-2-yl)-2,5-diphenyltetrazolium bromide (MTT), 5,50, 6,60-tetrachloro-1,10, 3,3-tetraethylbenzimidazolyl-carbocyanine iodide (JC-1), phenylmethylsulfonyl fluoride (PMSF) and trizma/hydrochloride (Tris/HCl) were obtained from Sigma-Aldrich (St. Louis, MO, USA). Dulbecco’s modified eagle medium (DMEM), dimethyl sulfoxide (DMSO), trypsin, dulbecco’s phosphate-buffered saline (DPBS), tissue culture-grade water, the ViaLight plus ATP assay kit and the Hanks balanced salt solution (HBSS) were obtained from Lonza (Walkersville, MD, USA). Fetal Bovine Serum (FBS) was purchased from Thermo Fisher Scientific (Waltham, MA, USA). The Oxiselect™ Intracellular ROS assay kit (Green Fluorescence) was purchased from Cell Biolabs (San Diego, CA, USA). XF Cell Mito Stress Kit was purchased from Agilent Technologies (Santa Clara, CA, USA). The GSH/GSSH-Glo^™^ assay kit and caspase-glo 3/7 were purchased from Promega, (Madison, WI, USA). The SOD activity assay kit (Abcam, Pretoria, SA, USA) was used. 7-Hydroxyflavanone was extracted from *Galenia africana* by BioPharm (Hamilton, New Zealand) and validated by means of high-performance liquid chromatography (HPLC)–ultraviolet using a reference standard obtained from Sigma-Aldrich (St. Louis, MO, USA).

### 4.2. In Vitro Models

**Figure 9:**
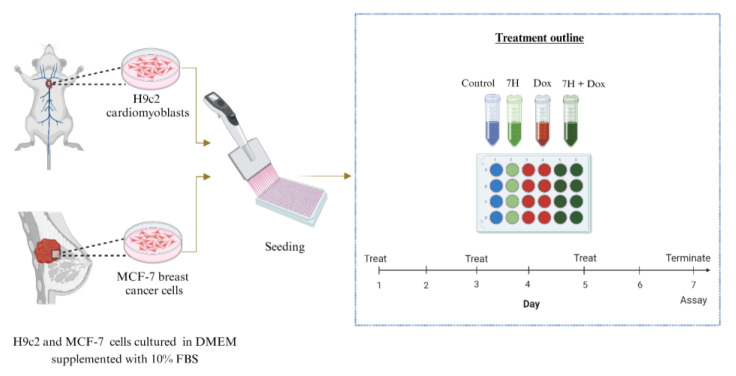
In vitro experimental set-up. Concisely, H9c2 cardiomyoblasts and MCF-7 breast cancer cells were treated every second day for 6 days. The cells were treated or co-treated with 7-Hydroxyflavanone in the absence or presence of Dox. After 6 days of treatment, biochemical assays were conducted.

Rat heart tissue-derived H9c2 cells and human metastatic breast cancer-derived MCF-7 cells were purchased from American Type Culture Collection (ATCC, catalogue number CRL-1446 and HTB-22, respectively). Briefly, H9c2 cells are frequently used in the initial screening process of new compounds and have also been used in models of cardiotoxicity. The MCF-7 cells process estradiol via cytoplasmic estrogen receptors, which makes them suitable models to study the efficacy of Dox. The cells were cultured using DMEM, supplemented with 10% FBS, and incubated under standard tissue culture (TC) conditions (37 °C, 95% humidified air and 5% CO_2_). A chronic model of DIC was mimicked by exposing the H9c2 cell to Dox (0.5 µM) for 6 days (Figure 9). To determine the therapeutic benefits of these flavonoids, cells were treated with 7H (1 µM) in the presence or absence of Dox (0.5 µM) for the same duration.

### 4.3. Dose Response

The effect of 7H was investigated on both cell lines to ensure that it did not potentiate cancer or induce adverse cardiac reactions. A dose screening of Dox, to attain the most desirable cytotoxic concentration, and 7H, to attain the most therapeutic dose, was conducted by measuring the cancer and cardiac cells’ metabolic status using the ViaLight plus ATP assay kit, per the manufacturer’s instructions. H9c2 cardiomyocytes and MCF-7 breast cancer cells were seeded (0.8 × 10^5^/mL) on 96-well plates (white, clear bottom) and then treated as described above. On the day of the assay, cells were lysed for 10 min and then 10 µL of the cell lysates were transferred into a new 96-well assay plate, containing BSA standards. The Bradford protein quantification assay was then conducted, and absorbance was read using SpectraMax^®^ i3x Multi-Mode Microplate Reader (Molecular Devices, San Jose, CA, USA). In the initial 96-well white plate, an ATP monitoring reagent (100 µL) was added to the cell lysates and then they were briefly incubated (5 min) before measuring luminescence as a quantification of ATP activity on SpectraMax^®^ i3x Multi-Mode Microplate Reader. Data were normalized to protein concentrations.

### 4.4. Oxidative Stress

The OxiSelect™ Intracellular ROS assay kit was used to measure cellular oxidative stress, per the manufacturer’s instructions. Briefly, H9c2 cells were seeded (1 × 10^5^/mL) in 24-well plates and treated as described above. The cells were then stained with 100 µL of DCFH-DA (2,7′dichlminorofluorescin diacetate, 20 µM) dye and then incubated for 30 min, under standard TC conditions. Cells were harvested via trypsinization, centrifuged at 300× *g* and collected into 2 mL Eppendorf tubes, before being resuspended in HBSS (100 µL). The activity of ROS was measured on the BD Accuri C6 flow cytometer (BD Biosciences, Franklin Lakes, NJ, USA).

To measure lipid peroxidation, H9c2 cells were first seeded (2 × 10^5^/mL) and treated in 6-well plates and then harvested per the methods described by Sangweni et al. (2020) [21]. Hereafter, the OxiSelect™ thiobarbituric acid (TBA) reactive substances (TBARS) kit was used to quantify the production of malondialdehyde, as a measure of lipid peroxidation, following the manufacturer’s instructions. Malondialdehyde production was quantified on SpectraMax^®^ i3x Multi-Mode Microplate Reader at an excitation and emission of 540 and 590 nm.

### 4.5. Inflammatory Cytokines

The R&D systems DuoSet ELISA kit (R&D Systems, Inc., Minneapolis, MN, USA) was used to quantify the secretion of inflammatory cytokine interleukin-6 (IL-6), per the manufacturer’s instructions. Concisely, supernatant media were collected from previously treated H9c2 cardiomyoblasts and then collected into 2 mL Eppendorf tubes. Prior to the assay, 96-well plates were coated with the respective capture antibody and then incubated at room temperature overnight. The next day, standards and assay samples were prepared in accordance with the manufacturer’s instructions. Optical density was determined using SpectraMax^®^ i3x Multi-Mode Microplate Reader set to a wavelength of 450 nm.

### 4.6. Endogenous Antioxidant Levels

The GSH/GSSG-Glo™ and colorimetric SOD activity assays (Abcam, Pretoria, SA, USA) were performed to measure the levels of endogenous antioxidant activity, per the manufacturer’s instructions. The cells were treated as previously mentioned in either 96-well (white, clear bottom, seeded at 0.8 × 10^5^/mL) or 24-well (seeded at 1 × 10^5^/mL) plates for the GSH/GSSG and SOD assays, respectively. For the SOD activity assay, cells were prepared as previously described by Sangweni et al. (2020) [21]. Superoxide dismutase activity was then measured at an absorbance wavelength of 450 nm on SpectraMax^®^ i3x Multi-Mode Microplate Reader. The relative luminescent unit (RLU) of GSH and GSSG was measured on SpectraMax^®^ i3x Multi-Mode Microplate Reader.

### 4.7. Mitochondrial Bioenergetics

Mitochondrial health was quantified using the XF Cell Mito Stress assay kit on the Seahorse XF96 extracellular flux analyzer per the manufacturer’s instructions and using optimized injection concentrations previously described by Sangweni et al. (2021) [33]. H9c2 cells were seeded (1 × 10^4^/well, at 80 µL media/well) in an XF 96-well microplate (Seahorse Bioscience) and treated as described above. On the day of the assay, cells were equilibrated for an hour in 180 µL of the base assay medium (supplemented with 10 mM glucose, 1 mM pyruvate and 2 mM glutamine) in a non-CO_2_ incubator. Mitochondrial modulators (1 µM oligomycin, 0.75 µM cyanide p-trifluoro-methoxyphenyl hydrazone (FCCP), 0.5 µM rotenone and antimycin A) were prepared as previously described [33]. Cell mito-stress was measured on the Seahorse XF96 extracellular flux analyzer (Seahorse Bioscience, Billerica, MA, USA). Subsequently, a Bio-Rad DC Protein assay was performed to normalize the data obtained. Data were expressed as pmol/min/mg protein and mpH/min/mg protein.

### 4.8. Mitochondrial Membrane Potential (MMP)

The JC-1 assay was performed to determine MMP per the manufacturer’s protocol. Cells that were previously seeded (0.8 × 10^5^/mL) in 96-well plates (black, clear bottom) were incubated with 100 µL of JC-1 dye (15.4 μM) for 45 min under standard TC conditions. Subsequently, relative fluorescence intensity, as a measure of MMP, was assessed on SpectraMax^®^ i3x Multi-Mode Microplate Reader, at an emission of 590 nm, for J-aggregates, and at that of 529 nm for JC-1 monomers. Mitochondrial images were captured on the Nikon inverted fluorescence microscope, where red fluorescence intensity represented high MMP, and green fluorescence indicated low MMP.

### 4.9. Evaluating Cell Death

Cell death was evaluated by measuring caspase 3/7 activity and the degree of apoptosis and necrosis, using the Caspase-Glo^®^ 3/7 luminescent assay kit and Annexin V-FITC (Invitrogen, Carlsbad, CA, USA) and propidium iodide (PI) dyes, respectively. For caspase 3/7 activity, H9c2 and MCF-7 cells previously seeded (0.8 × 10^5^/mL) in 96-well plates (white, clear bottom) were equilibrated to RT and then incubated in the dark with 100 μL of Caspase-Glo^®^ 3/7 reagent for 2 h on an orbital shaker (300–500rpm). Thereafter, caspase 3/7 luminescence activity was measured on SpectraMax^®^ i3x Multi-Mode Microplate Reader.

As a confirmation of cell death, cells were seeded in 6-well plates and then harvested using an in-house protocol [21]. H9c2 and MCF-7 cells were collected in 2 mL Eppendorf tubes and then stained with 1 µL of PI (2 μg/mL) and 1.5 µL of Annexin V prepared in 150 µL of staining buffer (DPBS supplemented with 10% FBS) before being incubated in the dark for 10 min, for the H9c2 cells, or 20 min, for the MCF-7 cells. Live, early, and late apoptotic and necrotic cells were detected on the BD Accuri C6 flow cytometer (BD Biosciences) using FITC signal detector FL1 (excitation = 488 nm; emission = 530 nm) for Annexin V-positive cells and the FL3 detector (excitation = 488 nm; emission = 670/LP) for PI-positive cells.

### 4.10. Western Blot Analysis

The expression of proteins involved in mitochondrial function, autophagy and cell survival were quantified by means of Western blot analysis. Concisely, H9c2 cell protein concentrations were quantified using the RC DC™ assay per the manufacturer’s instructions. Equal amounts of protein samples were separated using Criterion TGX stain-free protein midi gels (Bio-Rad Laboratories, Hercules, CA, USA) and then transferred to Trans-Blot Turbo Midi Nitrocellulose transfer packs. Membranes were blocked in either 5% (*w*/*v*) skim milk or bovine serum albumin (BSA) for 2 h and then incubated overnight with a primary antibody prepared in 2.5% BSA (phosphorylated protein kinase B (pAkt) (1:1000), phosphorylated 5′ adenosine monophosphate-activated protein kinase (pAMPK) (1:800), phosphorylated mammalian target of rapamycin (pMTOR) (1:1000), Beclin-1, phosphoinositide 3-kinases (PI3K) (1:1000), mTOR (1:1000), total Akt (1:1000) and peroxisome proliferator-activated receptor-gamma coactivator (PGC1-α)) on an orbital shaker at 4 °C. The following day, membranes were incubated with the respective secondary antibody for 1 h and then detected on a ChemiDoc Touch System using Image Lab Software 6.1 (Bio-Rad Laboratories, Hercules, CA, USA).

## 5. Conclusions

The results presented in this study demonstrate novel findings on the cardioprotective benefits of 7H when used as an intervention strategy against DIC. This was highlighted through the flavonoids’ ability to enhance endogenous antioxidant levels, thereby regulating mitochondrial bioenergetics, autophagy and apoptosis in an in vitro model of DIC. Additionally, 7H demonstrated a noticeable but insignificant effect on the efficacy of Dox when tested on the MCF-7 breast cancer cells. Taken together, these findings support the use of 7H as a potential cardioprotective candidate; however, further studies are needed to establish the clinical relevance of 7H’s therapeutic benefits on the heart, either as an adjunct to Dox or in other models of CVD. In addition, the current study also highlights the importance of using preclinical models that not only investigate the prophylactic benefits of cardioprotective compounds against DIC but also assess the effect that these compounds have on the efficacy of chemotherapeutic drugs using in vitro or in vivo cancer models.

## Figures and Tables

**Table 1:** Mitochondrial bioenergetics parameters (cardiac cells).

Metabolic Parameters (mpH/min/µg Protein)	Treatment			
	Control	7H	DOX	Dox + 7H
Basal respiration	48.1 ± 0.9	51.8 ± 2.1	15.4 ± 0.2 **	21 ± 0.7 *
Maximal respiration	116.3 ± 5.2	145.6 ± 5.7	16.7 ± 1.7 ***	40.5 ± 2.3 ***^, ##^
ATP-linked respiration	44.6 ± 0.9	46.9 ± 1.6	12.5 ± 0.2 ***	20.25 ± 0.6 **
Spare respiration	58.2 ± 1.0	93.83 ± 4.1	6.9 ± 0.7 ***	19.6 ± 2.9 ***^, #^
State apparent	2.6 ± 0.1	3.1 ± 0.1	3.1 ± 0.1	2.0 ± 0.1
Coupling efficiency	90.0 ± 1.6	83.9 ± 5.5	44.6 ± 0.9 ***	94.8 ± 1.1 ^###^
Respiratory control	47.9 ± 6.2	32.7 ± 5.0	12.7 ± 2.7 ***	32.9 ± 6.2 ^##^

Data represent mean ± SEM; *n* = 4. Significance is indicated as * *p* ≤ 0.05, ** *p* ≤ 0.01 and *** *p* ≤ 0.001 versus the control; ^#^ *p* ≤ 0.05, ^##^ *p* ≤ 0.01 and ^###^ *p* ≤ 0.001 versus Dox.

## Data Availability

Not applicable.
